# Improving Child Mental Health Policy in Canada

**DOI:** 10.7759/cureus.19974

**Published:** 2021-11-28

**Authors:** Ibraheem O Alimi, Ian Mathies, Arielle Archibald, Camille Compton, Emmanuel Keku

**Affiliations:** 1 School of Medicine, St. George’s University, St. George’s, GRD

**Keywords:** public mental health, pediatric mental health, child and adolescent psychiatry, child and youth mental health, health policy

## Abstract

Evergreen is Canada’s first official national mental health framework for children that was developed by the Mental Health Commission of Canada in 2010. The program is primarily an online consultation service, which is a beneficial aspect since it provides widespread access for those seeking mental health services for children, especially those in rural and underserved areas. Despite the program’s benefits and high ratings, Canada still lacks an adequate mental health framework for children because not all provinces and territories fulfilled the World Health Organization (WHO) criteria for child mental health, which shows that Evergreen has not been effective. As summarized in this review article, out of the 13 provinces and territories, the four provinces that met the minimum criteria for the WHO guidelines for child mental health policies were Ontario (ON), Alberta (AB), Saskatchewan (SK), and British Columbia (BC), with British Columbia being the leader in child mental health policies in Canada. For those that met the guideline, many performed poorly or failed to meet some of the WHO evaluation criteria for child mental health policies. For future progress, Canada should assess and evaluate its child mental health policies and incorporate that into a new and improved national standard and framework. Mental health data from Canada should also be analyzed to either implement an improved system or to fix old systems such as Evergreen that are currently in place. Finally, child mental health policy for Canada should constantly be reevaluated and improved to compensate for changes over time.

## Introduction and background

Approximately 1.1 million Canadian youths suffer from a mental health disorder, and this accounts for 14% of Canadian youths [1,2]. For many youths who have a mental health disorder, their conditions persist into adulthood, and the onset age for more than half of the cases is 14 years old [3]. As a result, this can influence the quality of life for the majority of the individual’s lifetime, and the conditions can worsen with greater severity if interventions are not made earlier on during child and adolescent development. Not only does the condition worsen with time, but they are also more likely to reoccur during adulthood if interventions are not made early during youth development. Due to the lifetime nature of mental illnesses, they are one of the costliest health conditions in Canada, resulting in economic losses and a total burden of more than $14.4 billion annually [4]. Untreated illnesses can also lead to increased rates of suicide, hospitalization, and substance abuse [5]. Canadian youths seeking services encounter several hurdles, namely, poor service access and long wait times extending up to one year [6]. Other countries have managed to develop successful national mental health policies for children, such as Australia with their Headspace program, Ireland with Jigsaw, and the UK with Youthspace [7,8]. Therefore, incorporating aspects of these strategies into the Canadian system along with the World Health Organization (WHO) guidelines and criteria could be a crucial component in creating an improved and effective national framework.

## Review

Critique of child mental health policy in Canada

Globally, the magnitude of mental health problems we face continues to rise and jeopardize the future of children in many countries. To address these issues, we need to start analyzing the challenges we are currently facing. This includes looking at the gaps in access to services within communities, the methods used to quantify mental disorders among youths, the economic costs, and the burden it can have on society. In Canada, there are many gaps in the policy process, ranging from the data-gathering capacity all the way to the resources needed to develop and implement programs and services within communities.

Kutcher et al. (2010) analyzed the policy process among 10 provinces in Canada using the WHO Assessment Instrument Tool for Mental Health Services (WHO-AMS) [5]. The WHO framework was used as it is currently the only internationally recognized and developed framework available. Overall, the results concluded that the provinces that followed the WHO template received mixed reviews regarding their policy or plan of action. This showed that there is a lack of cohesiveness among provinces when addressing child mental health issues. A comprehensive policy framework helps provide transparency and consistency between the government and community members to ensure quality services are provided; this is key in ensuring success when creating a policy. With the lack of a structured and complete framework, the roles and responsibilities of stakeholders are unclear, leading to miscommunication and hindering effective and efficient decision-making skills that can disrupt the operation and management of the policy [9].

An important question that should be asked is, “Why wasn’t a structured framework used to develop and implement policies in the other provinces?” After careful analysis, it is clear that the four provinces that met the WHO criteria had two things in common: large population size and a high incidence and prevalence rate of child mental health disorders [5]. In addition, these provinces received public backlash for their long waiting times to access services and high incidence of teen violence and disruptive behavior [5]. Given the stigma already associated with mental health disorders, this reactive approach by policymakers can worsen such stigma and misconceptions about mental health. Although these commonalities contributed to the development of a more comprehensive framework within these provinces/territories, does this mean that issues within other provinces should not be addressed in the same manner? The answer is no. Although strategies have been implemented to address key issues in the other provinces, such as suicide, substance abuse, and eating disorders, without a comprehensive plan in place, it is easy to misidentify and neglect areas of concern that can make it difficult to assess the effectiveness of the strategies [5].

According to Shakya et al. (2010), an estimated $23 billion is spent annually on medical bills, disability, and sick leaves in Canada; however, there was no budget dedicated exclusively toward child mental health policy in Canada [10]. For several years, Canada had failed to establish and implement a national child and adolescent mental health policy by which all provinces/territories must abide; this is unacceptable. Canada is currently facing many challenges regarding implementation, one being the lack of patient-oriented research being conducted to produce evidence that supports the implementation of efficient and cost-effective interventions. Limited funding has also impacted the ability of trained researchers to conduct these studies. By improving the research environment, it can address other ongoing challenges, such as the incorporation of child and adolescent mental health into the primary care settings. Currently, several approaches have been applied in these settings to help expand the roles of clinicians and nurses, in an effort to increase their mental health competencies to facilitate effective diagnosis, treatment, and management of mental disorders [10]. Unfortunately, these initiatives are still being developed, but with federal support, the likelihood of success both regionally and locally is inevitable.

The use of a multifaceted approach can help them make the necessary changes to properly address the needs of the population. The effects of child mental health services are not restricted to the healthcare sector because other agencies are affected by this issue and have a supporting role to play [10]. The education system is currently one that is primarily affected, and in recent times, it has been recognized as an important vehicle through which mental health promotion, mental disorder prevention, case identification, and continued care can be addressed.

Mental health is a crucial part of overall health; therefore, it should become a priority. Evergreen, which was developed in 2010 as the first national child and youth mental health framework in Canada, contains many strategies to help tackle the ongoing issues regarding child mental health [11].

Details about Canada’s child mental health policy

The article by Kutcher et al. (2010) described 13 territories in Canada and their individual policies for child mental health [5]; however, there was no overarching uniform framework in which all of Canada must abide prior to 2010. With this lack of structure, there was no nationally mandated set of criteria for the provinces to follow in order to ensure a minimum standard for child mental health [5]. Therefore, the federal government was not held accountable to assure access to acceptable support for children with mental health issues.

Currently, Canada’s mental health policy is most effective against adults and includes advocacy, promotion, and accessible interventions, which appear to have significant effects on that population. According to a study published by Stephens et al., there is a strong emphasis on mental health promotion and disease prevention [12]. Furthermore, the interventions focused on resilience and other methods that have resulted in “problem reduction and even prevention” [5]. Considering that these successful outcomes were published almost 20 years ago, it is strange that these methods have not been applied nationally to the children of Canada.

Typically, in developing countries that need assistance in certain areas, the World Health Organization (WHO) establishes guidelines to improve that country’s situation. Although Canada is a developed country, some areas have adopted this strategy by following the WHO criteria for child mental health in order to compensate for the federal government’s lack of action toward creating a national framework. Table 1 depicts the process steps that need to be taken by different provinces/territories toward aligning with the WHO guidelines. The results show that out of the 13 provinces/territories, only four of them fulfilled these WHO criteria: Ontario (ON), Alberta (AB), Saskatchewan (SK), and British Columbia (BC).

The lack of a coherent national benchmark for child mental health resulted in the establishment of a national youth mental health policy framework called Evergreen, which was developed in 2010. Evergreen, Canada’s first national framework for child mental health, utilizes an Internet-based approach for child mental health consultations [11]. Although it has not yet been proven to be successful and effective, it has raised awareness of the issue and has high approval among the citizens of Canada.

**Table 1:** WHO steps and guidelines for developing a child mental health policy. AB–YT: official postal abbreviations for Canadian provinces/territories; N/A: not available. Adapted from Kutcher et al., (2010) [5].

Guidelines and Steps	Provinces and Territories												
	AB	BC	MB	NB	NL	NS	NT	NU	ON	PE	QC	SK	YT
1) Gather information and data for policy development	Yes	Yes	No	No	No	No	No	No	Yes	No	Yes	Yes	No
2) Gather evidence for effective strategies	Yes	Yes	No	No	No	No	No	No	Yes	No	Yes	Yes	No
3) Undertake consultation and negotiation	Yes	Yes	No	No	No	No	No	No	Yes	No	Yes	Yes	No
4) Exchange with other countries	N/A	N/A	No	No	No	No	No	No	N/A	No	N/A	N/A	No
5) Develop the vision, values, principles, and objectives of the policy	Yes	Yes	No	No	No	No	No	No	Yes	No	No	Yes	No
6) Determine areas for action	Yes	Yes	No	No	No	No	No	No	Yes	No	Yes	Yes	No
7) Identify the major roles and responsibilities of different stakeholders and sectors	Yes	Yes	No	No	No	No	No	No	Yes	No	Yes	Yes	No

Analysis and evaluation of Canada’s child mental health policy

Although Evergreen has been developed, it is yet to be implemented effectively on a national scale. Effective implementation on a national level allows individual provinces and territories to customize the policy to best suit their demographics. The absence of a national consensus and collaborative policy development has resulted in splintered mental health delivery for Canada’s children. Individual provincial-level policies have resulted in several smaller initiatives lacking the impact and effectiveness necessary to provide enhanced pediatric mental health care [5].

Upon analysis and evaluation, only three of the four provinces that met the WHO criteria made excellent financial provisions toward their policy as shown in Table 2 [5]. Ontario acknowledges their shortcomings, citing the lack of an effective national policy and clear standards provides little financial accountability and little incentive toward improving child mental health services [13]. This is important as insufficient funding can lead to increased long-term financial expenditures to address consequences such as increased rates of hospitalizations, prevalence, and incidence of child mental health disorders [5].

As shown in Table 2, intersectoral collaboration was excellently executed in all three provinces except for Ontario, which received a poor score. Collaboration with other sectors such as the Departments of Social Services, Education, and Corrections is paramount in addressing Canada’s pediatric psychiatric service gap. All stakeholders must participate in consistent communication to effectively monitor and enhance the quality of child mental health services.

As displayed in Table 2, all four provinces received neutral performance ratings regarding legislation and human rights. They lacked definite legislation outlining the consent and confidentiality of child mental health services, with the tendency to default to the common law “mature minor” rule [14]. Canadian pediatric mental health policy must develop clear national guidelines for minors seeking services that ensure that they are confidentially in accordance with national and international laws. It must also maintain WHO human rights standards while respecting the autonomy and dignity of all minors.

Table 2 also shows that all provinces failed to meet WHO’s information systems standards. A thorough evaluation must therefore be implemented to assess the current information system and identify necessary areas for improvement, and methods for allowing data obtained to be effectively utilized to inform policies [15]. Strengthening of information systems is strongly recommended as this will be essential to accessing child mental health services, the effectiveness of consultations, and the equitable distribution of services throughout Canada.

As shown in Table 2, the four areas of advocacy, research and evaluation of policies and services, organization of services, and promotion, prevention, treatment, and rehabilitation were very well executed across all provinces [5]. Each of the four provinces followed and met the recommended WHO criteria for each of these categories.

British Columbia was the only province to achieve excellent quality improvement as displayed in Table 2, while the other provinces poorly complied with the WHO standards [5]. Their policy acknowledged the importance of quality improvement evaluation on policy formation by implementing several continuous quality improvement projects to assess their performance since 2007. Similar strategies should be undertaken nationally by other provinces to assess the standard of services provided, thus allowing accountability for service quality and evaluation of cost-effective strategies [15].

Table 2 also shows that all provinces performed poorly in following the WHO “Improving Access and Use of Psychotropic Medicines” guidelines [16]. While Canada has increased its general usage of psychotropic medications in recent years, little has occurred to improve access to more effective medications [17]. Several new and more effective psychotropic medications have not been granted Canadian regulatory board approval, thus making them unavailable and resulting in increased rates of pediatric medications being prescribed for “off-label” purposes [17]. This is concerning as the misuse of these medications comes with significant side effects such as affecting cardiovascular functioning and overall child development [17]. Canada must ensure timely review of new medications for regulatory approval and outline better policies for “off-label” prescription use.

In addition to psychotropic medications, human resources development and training was another area of concern, with all provinces scoring neutral ratings and failing to define methods for evaluating personnel (Table 2). Considering that a major barrier to pediatric psychiatric health services is the shortage of personnel, we recommend the creation of a national plan addressing this and increasing the recruitment of child mental health personnel and specialists [18]. Access to subspecialized psychiatric services is also another challenge. Therefore, plans should also ensure that subspecialized training is available and accessible to personnel [18].

**Table 2:** WHO evaluation criteria for child mental health policies. AB–YT: official postal abbreviations for Canadian provinces/territories. Adapted from Kutcher et al., (2010) [5].

Evaluation Criteria Areas	Provinces and Territories			
	AB	BC	ON	SK
Financing	Excellent	Excellent	Neutral	Excellent
Intersectoral collaboration	Excellent	Excellent	Poor	Excellent
Legislative and human rights	Neutral	Neutral	Neutral	Neutral
Advocacy	Good	Good	Excellent	Good
Information systems	Fail	Fail	Fail	Fail
Research and evaluation of policies and services	Good	Excellent	Good	Good
Quality improvement	Poor	Excellent	Poor	Poor
Organization of services	Good	Good	Good	Good
Promotion, prevention, treatment, and rehabilitation	Excellent	Excellent	Good	Excellent
Improving access to and use of psychotropic medicines	Poor	Poor	Poor	Poor
Human resources development and training	Neutral	Neutral	Neutral	Neutral

Implementation of an improved child mental health policy in Canada

As previously mentioned under Canada’s child mental health policy details, analysis, and evaluation, only four provinces have a youth mental health plan that fulfills WHO criteria, which suggests that Evergreen, Canada’s first national policy on child mental health, is not being implemented effectively. For the policy to be effective at improving youth mental health status in Canada, it must fulfill four main steps: 1) development of an improved national policy that fulfills the WHO criteria for child and adolescent mental health policies and plans, 2) adoption of the national policy by all provinces and territories across Canada, 3) implementation of the national youth mental health policy, and 4) evaluation of the national child and adolescent mental health policy [16].

The first step, which is the development of an improved national policy that fulfills the WHO criteria for child and mental health policies and plans, ensures that the national policy is an effective policy that meets international standards. The WHO (2005) summarize the seven criteria in which an effective policy must be fulfilled during the developmental process: 1) gathering information and data for policy development; 2) gathering evidence for effective strategies; 3) undertaking consultation and negotiation; 4) exchanging with other countries; 5) developing the vision, values, principles, and objectives of the policy; 6) determining areas for action; and 7) identifying the major roles and responsibilities of different stakeholders and sectors [16].

Once this step has been completed, the improved national policy will then need to be adopted by all the provinces and territories across Canada. It is important that provinces such as Ontario, Saskatchewan, Alberta, and British Columbia that already have a youth mental health policy that meets WHO criteria also adopt the improved national policy, as that will reduce fragmentation and also ensure that a uniform and cohesive youth mental health policy exists across the whole country. Once the policy has been adopted by all the provinces and territories across Canada, it then needs to be implemented on a local scale in order to improve the mental health status of Canadian children and youths. It is also important that the national policy adopted by each province be tailored to fit the needs of each community before it is implemented on a local level.

Once the policy has been implemented, it needs to be continuously monitored and evaluated using the 11 WHO evaluation criteria for child mental health policies as displayed in Table 2 and Funk et al. (2011) [15]. Evaluation and monitoring of the national mental health policy are important because it helps determine the strength of the policy and the areas that can be improved over time. As long as the national policy fulfills each of these evaluation criteria with excellent ratings, there should be a gradual improvement in the status of child mental health in Canada.

## Conclusions

Taken together, although Evergreen, Canada’s first official national mental health framework for children, has been developed, significant improvements are still required to meet WHO guidelines and criteria. Out of the 13 provinces and territories, only four provinces met the WHO guidelines for child mental health policies, which shows that Evergreen has not been effective. For the provinces that meet the WHO guidelines, many perform poorly or fail to meet some of the WHO evaluation criteria for child mental health policies. To maintain progress, Canada should develop an improved national child mental health policy that fulfills both the WHO guidelines and evaluation criteria for child mental health policies. This improved policy should be incorporated into a national standard and adopted by all provinces and territories in order to ensure cohesiveness and unity and reduce fragmentation. Once it has been adopted by all provinces and territories, it should be implemented effectively and constantly reevaluated and improved to compensate for changes in the local communities and society.
